# Ophthalmic Therapeutics for the General Practitioner

**Published:** 1900-07

**Authors:** A. W. Calhoun

**Affiliations:** Atlanta, Ga.


### OPHTHALMIC THERAPEUTICS FOR THE GENERAL PRACTITIONER.

BY A. W. CALHOUN, M.D., LL.D.,
ATLANTA, GA.

Chemistry is constantly presenting so many new drugs to the physician's attention, that it is almost confusing, and certainly difficult, to test and decide which should be retained. However, there are some that deserve to be held in high esteem by the specialist and general practitioner.

Among the newer drugs useful in ocular therapeutics we have four that are particularly valuable and to which I wish to direct your attention.

1. *Protargol.*—This brown-colored proteid compound, containing about eight per cent. of silver, is considered better than any inorganic silver salt yet used. It is much less painful and less dangerous to the epithelium, while it penetrates deeper than the silver nitrate, and on this account can be used much more freely and frequently.

One of the great objections to the nitrate is the long-continued and violent pain following its application to the conjunctival surface.

There is but little pain from the Protargol, and it can be applied several times a day if needful. It has been used in phlyctenular ophthalmia and injected into the lacrimal sac in dacryocystitis with great benefit; and quite recently is being applied to the middle ear in suppurative inflammations with satisfactory results. Perhaps in gonorrheal conjunctivitis nothing can replace the silver nitrate; but in all other inflammations and irritations of the ocular mucous membrane, even ophthalmia neonatorum, Protargol in five to ten per cent. watery solution is much better for the general practitioner. It must be borne in mind that the too free use of even this mild silver salt will produce argyrosis, the dirty gray or blue-black discoloration of the conjunctiva we all know so well; therefore care must be exercised in the use of it.

Although Protargol is not the absolutely ideal agent in the treatment of inflammatory mucous membrane affections of the eye, it is in many respects an important advance upon the silver nitrate.

2. *Extract Suprarenal Capsule.*—The local and constitutional effects of this preparation are very remarkable, and it is much used both by the general practitioner and specialist; indeed, it is surprising that it is not much more employed in general practice.

It is an unstable powder, has a brown color and a decidedly animal odor, absorbs moisture when exposed to the air, is weakened by heat and easily spoils in the presence of water. When needed it should be freshly prepared, as it has no well-defined solubility, mixing about five grains with one drachm of water. Usually it is filtered, but I use it without filtering, which is a tedious process; and, besides, I get also the benefit of applying at the same time the undissolved powder with the watery solution.

Experiments have shown that it is much more astringent than hemostatic, being perhaps the most powerful astringent known. The rapid cures of inflammation and the relief of pain from its use are due solely to its astringent property, as it is not an anesthetic, nor has it any antiseptic powers. Applied to the eye, the conjunctiva of the ball and lids becomes whitened in one or two minutes, but it has no effect upon the pupil, nor does it produce any constitutional effect when applied to the mucous surfaces.

In making operations upon the eye or nose, it is best to precede the application of the extract by that of a four or five per cent. solution of cocain, so as to get the combined effects of the anesthetic and the astringent. For years cocain has rendered almost painless the various operations upon the eye, the ear, nose and throat, but the accompanying hemorrhage is frequently annoying and very embarrassing. Under the influence of the two drugs, not mixed but one following the other, both pain and hemorrhage are absent. Within the last week or two I have seen its wonderful astringent effect in two cases of nasal hemorrhage. In both cases the bleeding had continued for several days, and all the remedies used had failed to check it. After clearing away the blood-clots and bringing into view the bleeding surfaces, the hemorrhage ceased almost instantly upon the application of the suprarenal extract. A few treatments sufficed to effect a perfect cure. It is now being used with great advantage in operations and applications of the galvano-cautery upon the eye, ear, nose, throat, urethra and other mucous surfaces, not only for its profound hemostatic effect, but to enhance the local anesthesia from cocain, holocain, etc. Its prompt and efficient astringent effect would suggest the danger of secondary hemorrhage after operations, and it would be well to guard against such a possibility. It has been demonstrated to be also a heart tonic, and its physical properties would point to its usefulness in cardiac failure and other diseases of the heart of a varied character.

3. *Holocain.*—This is another of the new remedies that has come to stay; but its chief use is as a local anesthetic in ophthalmic surgery. It is one of those new synthetic substances, is soluble in cold water, and should be applied in one per cent. solution. Wherever cocain is indicated the holocain can also be used—perhaps to greater advantage, as it does not dilate the pupil nor disturb the accommodation. It acts quickly and causes no pain. The corneal epithelium is affected less by it than by cocain, and its use is followed by much less hyperemia. It is decidedly antiseptic in its action. As it is a systemic poison, it must not be injected hypodermically, nor be applied to other than the ocular mucous membranes. Holocain does not primarily contract the blood-vessels, and therefore does not tend to control hemorrhage, as is the case with cocain; but by those who have had much experience with it, it is considered not only as good as cocain, but preferable to it.

4. *Euphthalmin.*—The chief claim of this new remedy to the attention of the oculist and general practitioner is as an agent for quickly dilating the pupil. Every day we see cases where it is desirable to dilate the pupil for ophthalmoscopic examination. The objection to most of these dilating agents is the annoying length of time after their use that it takes for the pupil to contract to its normal size.

In a few minutes after the instillation of a five to ten per cent. watery solution of euphthalmin the pupil is widely dilated and ready for the ophthalmoscopic examination. In about twenty-four hours the pupil has regained its normal size. It is purely a mydriatic and does not tend to increase the tension of the ball. It produces no hyperemia of the conjunctiva, and does not roughen the cornea. Its chief recommendation is for routine use to dilate the pupil for ophthalmoscopic examination, and, secondarily, as a substitute for atropia in cases of intolerance of that drug.

